# Salt tolerance of a wild ecotype of vetiver grass (*Vetiveria zizanioides* L.) in southern China

**DOI:** 10.1186/s40529-016-0142-x

**Published:** 2016-10-06

**Authors:** Wan-gou Liu, Jin-xiang Liu, Mei-ling Yao, Qi-fu Ma

**Affiliations:** 1 Life Science and Technology School, Lingnan Normal University, Zhanjiang, 524048 People’s Republic of China; 2 School of Veterinary and Life Sciences, Murdoch University, 90 South Street, Murdoch, WA 6150 Australia

**Keywords:** Wild vetiver grass (*Vetiveria zizanioides* L.), Salt stress, Water relation, Antioxidant enzymes, Photosynthetic rate, Growth

## Abstract

**Background:**

Vetiver grass (*Vetiveria zizanioides* L.) is widely used in more than 120 countries for land management (e.g. rehabilitation of saline lands). A wild ecotype of vetiver grass was found in southern China in the 1950s, but little is known about its adaptability to saline stress. For the purpose of understanding its tolerance to salinity as well as corresponding tolerance mechanisms, in a greenhouse with natural lighting, seedlings were grown in culture solutions and subjected to a range of NaCl concentrations for 18 days.

**Results:**

Compared to no NaCl treatment, 200 mM NaCl significantly reduced leaf water potential, leaf water content, leaf elongation rate, leaf photosynthetic rate and plant relative growth rate and increased leaf malondialdehyde (MDA) content, but the parameters showed only slight reduction at 150 mM NaCl. In addition, salinity caused an increase in the activity of antioxidant enzymes in leaves. Moreover, increasing NaCl levels significantly increased Na^+^ but decreased K^+^ concentrations in both roots and leaves. The leaves had higher K^+^ concentrations at all NaCl levels, but lower Na^+^ concentrations compared to the roots, thereby maintaining higher K^+^/Na^+^ ratio in leaves.

**Conclusions:**

Our results showed that the salinity threshold of this wild vetiver grass is about 100 mM NaCl, i.e. highly tolerant to salt stress. This wild vetiver grass has a high ability to exclude Na^+^ and retain K^+^ in its leaves, which is a critical strategy for salt tolerance.

## Background

Salinity is a one of the major environmental stress with over 800 million ha of land globally are salt-affected, causing great losses in agriculture productivity (Ledesma et al. 2016). One approach to increase the use of saline lands is to identify new plant species with salt tolerance. To date, biotechnology has not yet developed salt tolerant cultivars for agriculture use (Himabindu et al. 2016) probably for the reason that salt tolerance is a complex trait determined by many genes which interact strongly with environmental factors (Munns et al. 2012). Therefore, the development of salt tolerant plants depends mainly on screening plant species with high salt tolerance and understanding the tolerance mechanisms (Feng et al. 2014).

Vetiver grass (*Vetiveria zizanioides* L.) is a perennial graminaceous plant native to tropical and subtropical areas (Ghotbizadeh and Sepaskhah 2015). This species is distinguished by its strong and extensive root system which can descend 5 m under tropical conditions. The extensive, thick and deep root system with a tensile strength equal to 1/6th that of mild steel provides the plant with a superior advantage for adaptation to a wide range of ambient stresses, for example drought, flood, extreme temperatures, heavy metals, acidity and alkalinity, and salinity (Ghotbizadeh and Sepaskhah 2015; Truong et al. 2002; Zhou and Yu 2009). In rain-fed agriculture the deep-rooted perennial grasses in rotation with annual crops may help recover the balance between water use and rainfall, therefore, preventing rising water tables taking salts to the surface (Munns and Tester 2008). Vetiver grass is already widely used for saline land rehabilitation (Datta et al. 2011; Donjadee and Tingsanchali 2012).

Most vetiver genotypes flower but do not produce seeds. In Wuchuan County (21°30′N, 110°50′E), Guangdong Province of China, a wild and fertile ecotype of vetiver grass was found in May, 1957 (Xia and Ao 1998). This vetiver community extended over an area of about 7000 ha in the 1950s, but has now completely disappeared in the wild due to land clearing. Fortunately, the germplasm of this species was introduced into the Grass Research Station of Lingnan Normal University in 2002 (Liu and Chen 2002) where it has since grown with the climatic conditions similar to that of its origin. The original community was located at an alluvial plain near the estuary of South China Sea, where the whole community was usually inundated during the rainy season (from April to September), but the ground water level sometimes might be 2 m deep during the dry seasons (from October to March) (Xia 2002). The repeated drying/rewetting cycles would raise the salinity of soil, leading to an expectation of salt tolerance by this vetiver ecotype. However, there have been no detail studies about its physiological responses under different environmental stresses. Understanding the stress physiology of this vetiver ecotype would not only help describe its biological characteristics, but evaluate its potential for applications in land management.

Salinity influences plants in two ways: (1) high salinity in the soil make it more difficult for roots to extract water, and (2) high concentrations of salt within the plant can be toxic (Munns and Tester 2008). In response to salinity, plants have evolved various mechanisms to mitigate osmotic stress, such as by stomatal closure to reduce water loss, by exclusion of Na^+^ from leaf to minimize the toxicity of Na^+^ and by sequestration of Na^+^ into root and leaf vacuoles to alleviate ionic stress to cytoplasm. The ability to exclude Na^+^ and to maintain low tissue Na^+^/K^+^ ratio is an essential aid for plant salt tolerance (Munns et al. 2012). Moreover, salt stressed plants can synthesize antioxidants in cells such as superoxide dismutase (SOD), catalase (CAT) and various peroxidases (POD) as scavengers of reactive oxygen species (ROS) (Wang et al. 2014). Although vetiver grasses are widespread in salt-affected regions, only a few studies have reported that they are moderately salt tolerant (Cuong et al. 2015; Zhou and Yu 2009) and the underlying mechanism of salt tolerance in vetiver is still unclear. In this study, we treated a wild ecotype of vetiver grass from Wuchuan County with a range of NaCl levels to assess its capacity for salt tolerance in terms of water and ion relations, leaf gas exchange, plant growth and antioxidant enzyme activity. The possible mechanism of salt tolerance by this ecotype is discussed.

## Methods

### Plant material and growth conditions

Seeds of a wild ecotype of vetiver grass (*Vetiveria zizanioides* L.) were collected in October 2012 from the Grass Research Station of Lingnan Normal University, Zhanjiang, Guangdong province China (20°55′N, 110°11′E). Its 1000 seed weight was 291 mg. Seeds were sown in soil in pots (18 cm high, 25 cm in diameter). Seven months after sowing, the seedlings (about 30 cm high) were transplanted into plastic buckets (depth 27.5 cm, diameter 30 cm) containing Hoagland solution [2.5 mM Ca(NO_3_)_2_, 2.5 mM KNO_3_, 1 mM MgSO_4_, 0.5 mM KH_2_PO_4_, 45 μM Fe-EDTA, 23 μM H_3_BO_3_, 4.55 μM MnSO_4_, 0.16 μM CuSO_4_, 0.38 μM ZnSO_4_, 0.28 μM H_2_MoO_4_] and grown in a naturally-lit greenhouse. Nine months after sowing, uniform plants (about 60 cm high) were divided into four NaCl treatment groups (see below) with 60 plants per treatment, for a total of 240 plants. The plants were supported by a foam board while the roots were dipped into a 30 L Hoagland solution in a plastic container (length 50 cm, width 38 cm, depth 22.5 cm), and 15 plants were grown in each container.

The salt treatments included NaCl levels of 0, 100, 150 and 200 mM and each treatment was replicated four times in a randomized block design. To avoid osmotic shock (Albert 1975), NaCl levels were increased by 50 mM daily until the final levels of individual treatments were reached. The solutions were aerated for 2 h daily using aquatic pumps and replaced weekly. The pH of the nutrient solutions was adjusted to 6.5 ± 0.1 every day with 1 M KOH or 1 M H_2_SO_4_, as required. The experiment was completed after 18 days when plant growth was obviously suppressed by 200 mM NaCl but there was a lesser effect on plant by 100 and 150 mM NaCl, compared with no NaCl treatment. At harvest, 15 plants in every container were composited as one replication, i.e. 60 plants in four replications for each NaCl treatment.

### Measurement of leaf gas exchange

Before the plants were harvested for dry weight at the end of NaCl treatments, the third youngest fully expanded leaves were measured for net photosynthetic rate (*P*_*n*_), stomatal conductance (*g*_*s*_), transpiration rate (*E*), and intercellular CO_2_ concentration (*C*_*i*_) with a portable photosynthesis system LI-6400XT (LI-COR Inc., Lincoln, NE, USA). Five random measurements of 15 plants per container were composited as one replicate, and four replicates were taken between 9:00 a.m. and 12:00 noon under the conditions of photosynthetically active radiation of 1600 µmol m^−2^ s^−1^ via internal light source, leaf temperature of 35 ± 1 °C, relative humidity of 60 ± 5 %, and ambient CO_2_ concentration of 389 µmol mol^−1^ (*C*_*a*_). The stomatal limitation value (*L*_*s*_) was calculated as: (Ma et al. 2012)

$$L_{s} = 1 - C_{i} /C_{a} .$$

### Water relation measurement

At the end of NaCl treatment, leaf water potentials (*ψ*_*w*_) of the third youngest fully expanded leaves were measured from 15 plants per container as one replicate, with four replicates in each treatment. The measurements were taken using a pressure chamber (Model 1000, PMS Instrument, Albany, OR, USA) at the time 10:00–11:00 am.

After fresh weights (FW) of the roots and leaves were recorded, they were oven-dried at 105 °C for 15 min and then dried at 75 °C for 48 h and dry weights (DW) were recorded. Tissue water content (*WC*) was calculated on a fresh weight basis,

$${\text{WC }}\left( \% \right) = \left( {{\text{FW}}{-}{\text{DW}}} \right)/{\text{FW}} .$$

### Antioxidant enzyme activity assays

About 0.5 g of the youngest fully expanded leaves were ground in liquid nitrogen with a mortar and homogenized in 1 mL of 50 mM phosphate buffer (pH 7.0) containing 3 μM EDTA and 1 % polyvinylpolypyrrolidone (PVP). The homogenates were centrifuged at 12,000 rpm for 30 min at 4 °C and the supernatant was collected and used for antioxidant enzyme activity analysis.

Leaf SOD was measured through the inhibition of nitro blue tetrazolium (NBT) reduction with the ${\text{O}}_{2}^{{ - }}$ generated by the xanthine oxidase system. One unit (U) of SOD was defined as the amount of enzyme required to inhibit NBT reduction by 50 % under the assay conditions. The reduction of NBT was determined from an initial absorbance change using spectrophotometer at 560 nm after addition of xanthine oxidase at 25 °C. CAT activity was measured according to the method of Aebi (1984) by determination the disappearance of H_2_O_2_ by measuring the decrease in an absorbance at 240 nm of a reaction mixture containing 25 mM phosphate buffer (pH 7.8), 10 mM H_2_O_2_ and enzyme. One unit of CAT was defined as the amount of enzyme required to decrease absorbance by one per minute. POD activity was measured by the increase in absorbance at 470 nm due to guaiacol oxidation. The reaction mixture contained 25 mM phosphate buffer (pH 6.0), 0.05 % guaiacol, 10 mM H_2_O_2_ and enzyme. One unit of POD was defined as the amount of enzyme required to increase absorbance by one per minute. All the activities of SOD, CAT and POD were expressed as enzyme units in a dry weight basis (U g^−1^ DW). All the measurements were repeated four times.

### Lipid peroxidation assay

Lipid peroxidation was determined by measuring malondialdehyde (MDA) formation using the thiobarbituric acid method (Tang 1999). The fourth top leaf samples (1 g) were ground in liquid nitrogen and homogenized into 10 mL of 10 % trichloroaceticacid. After centrifugation at 4000 rpm for 10 min, 2 mL of supernatant was combined with 2 mL of 0.6 % thiobarbituric acid, heated in boil water for 15 min and cooled rapidly on ice. The mixture was then centrifuged at 4000 rpm for 10 min, and its absorbance was determined at 450, 532 and 600 nm with a UV-1600 spectrophotometer. The MDA concentration was estimated using the following formula, and MDA content was expressed in a dry weight basis (nmol g^−1^ DW).

$${\text{C }}\left( {\upmu {\text{M}}} \right) = 6.45\left( {{\text{A}}_{ 532} - {\text{A}}_{600} } \right) - 0.56 {\text{A}}_{ 450}$$

### Growth measurement

Before the commencement of the four NaCl treatments, the uppermost-leaf length (L_0_) was measured and plant initial dry weight (DW_0_, included shoot and root) was estimated by destructively oven-drying ten additional plants at 75 °C for 48 h. At the end of the NaCl treatments, the corresponding leaf length (L_1_) was measured again and plants were oven-dried for dry weight (DW_1_) by taking the average of 15 plants per container as one replicate.

Leaf elongation rate (*LER*, mm d^−1^) was calculated using the formula, $\left( {{\text{L}}_{ 1 } - {\text{L}}_{0} } \right)/t$, where *t* is the time interval (days).

Relative growth rate (*RGR*, mg g^−1^ d^−1^) was calculated using the formula, $\left( {{\text{lnDW}}_{ 1} - {\text{nDW}}_{0} } \right)/t$, in which *t* is the time interval (days) (Poorter 2001).

### Assay of Na^+^ and K^+^

The harvested plants were washed firstly with tap water and then distilled water, and the roots and leaves were separated. After oven-dried at 75 °C for 48 h, the samples were ground and passed through a 2-mm mesh sieve. The concentrations of Na^+^ and K^+^ in leaves and roots were measured following the previous method (Song et al. 2009) with some modifications. In brief, 0.2 g samples were processed in a muffle oven at 200 °C for 30 min and then 550 °C for 24 h, and the ash was dissolved in 0.2 mL of concentrated nitric acid. The volume of the extraction was adjusted to 100 mL with double distilled water, and the concentrations of Na^+^ and K^+^ were measured using a flame photometer (Flame Photometer 420, Sherwood Scientific Ltd, Cambridge, UK). The ability of ion selective transportation (*S*_*k/Na*_) was calculated from the following formula: (Zhou and Yu 2009)

$$S_{k / Na} = \left( {{\text{K}}^{ + } /{\text{ Na}}^{ + } {\text{in leaf}}} \right) / \left( {{\text{K}}^{ + } /{\text{ Na}}^{ + } {\text{in root}}} \right).$$

### Statistical analysis

All data in response to four NaCl treatments were subjected to one-way analysis of variance. Treatment differences were determined by Duncan’s multiple range test at *p* < 0.05 level. The statistical analyses were conducted using SPSS18.0 for windows (SPSS Inc., Chicago, IL, USA).

## Results

### Water relations

**Fig. 1:**
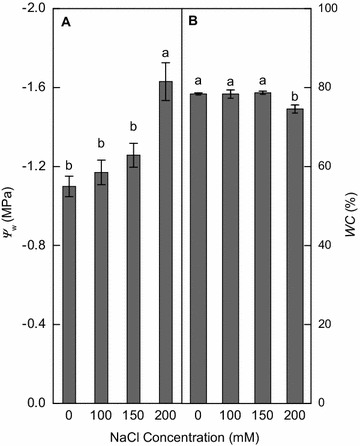
Effects of 0, 100, 150 and 200 mM NaCl for 18 days on **A** leaf water potential (*ψ*_*w*_) and **B** leaf water content (*WC*) of wild vetiver grass. Means (±SE n = 4) followed by *different letters* differ at *p* < 0.05

After vetiver plants were treated with NaCl for 18 days, leaf water potentials (*ψ*_*w*_) and content (*WC*) were similar among treatments of 0, 100 and 150 mM NaCl, but decreased significantly at 200 mM NaCl (Fig. 1A, B).

### Plant growth

**Fig. 2:**
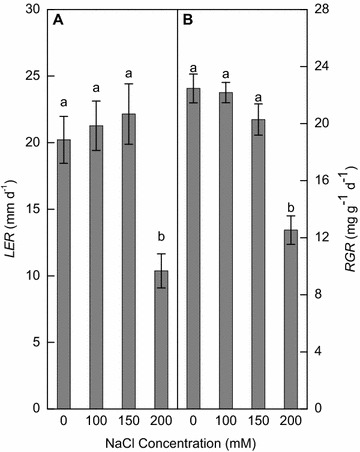
Effects of 0, 100, 150 and 200 mM NaCl for 18 days on **A** uppermost-leaf elongation rate (*LER*) and **B** plant relative growth rate (*RGR*) of wild vetiver grass. Means (±SE n = 4) followed by *different letters* differ at *p* < 0.05

During the period of NaCl treatments, the uppermost-leaf elongation rate (*LER*) increased slightly at 100 mM NaCl (5.2 %) and 150 mM NaCl (9.6 %), but significantly decreased at 200 mM NaCl (48.7 %) (Fig. 2A). Consistently, the relative growth rate (*RGR*) was reduced by 1.3, 9.7 % at 100, 150 mM NaCl, respectively, and 200 mM NaCl decreased *RGR* by 44.2 % (*p* < 0.05) compared with the no NaCl treatment (Fig. 2B).

### Leaf gas exchange

**Fig. 3:**
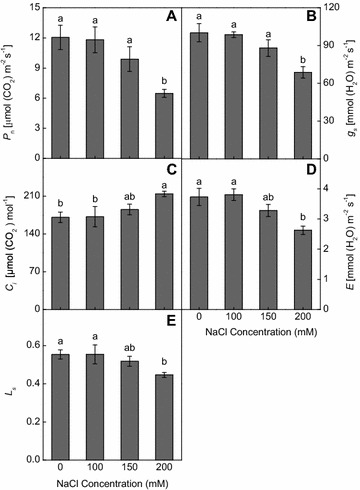
Effect of 0, 100, 150, 200 mM NaCl for 18 days on **A** the net photosynthetic rate (*P*_*n*_), **B** stomatal conductance (*g*_*s*_), **C** intercellular CO_2_ concentration (*C*_*i*_), **D** transpiration rate (*E*) and **E** stomatal limitation value (*L*_*s*_) of wild vetiver grass. Means (±SE n = 4) followed by *different letters* differ at *p* < 0.05

With increasing NaCl levels, leaf *P*_*n*_, *g*_*s*_ and *L*_*s*_ declined and significant reduction was observed at 200 mM NaCl (Fig. 3A, B, E). Conversely, leaf *C*_*i*_ was increased at 100, 150 and 200 mM NaCl by 0.7, 8.5 and 25.4 % respectively (Fig. 3C). Leaf *E* was highest at 100 mM NaCl and slightly decreased at 150 mM NaCl, but was significantly lowered at 200 mM NaCl (Fig. 3D).

### Antioxidant enzyme activities

**Fig. 4:**
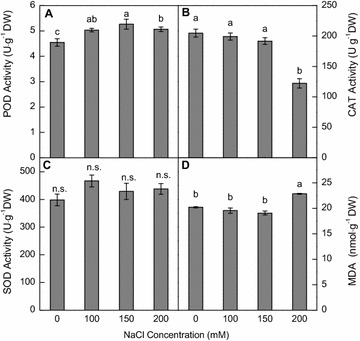
Effects of 0, 100, 150, 200 mM NaCl for 18 days on **A** POD, **B** CAT, **C** SOD activities as well as **D** MDA content of wild vetiver grass. *ns* no significance. Means (±SE n = 4) followed by *different letters* differ at *p* < 0.05 level

After NaCl treatments for 18 days, the activities of leaf POD at 100, 150 and 200 mM NaCl levels were significantly higher than at no NaCl level (Fig. 4A). In contrast, leaf CAT activities decreased with increasing NaCl levels, and significantly reduced at 200 mM NaCl (Fig. 4B). Although the peak SOD activity was observed at 100 mM NaCl, there were no significant differences among the NaCl treatments (Fig. 4C). Overall, this wild ecotype of vetiver grass maintained high activity of protective enzymes under saline condition.

### MDA content

Lipid peroxidation in leaves was responsive to NaCl levels, showing a significant increase at 200 mM NaCl but there were no significant changes at 100–150 mM NaCl, compared with the no NaCl treatment (Fig. 4D).

### Na^+^, K^+^ accumulation and ability of ion selective transportation (S_k/Na_)

**Fig. 5:**
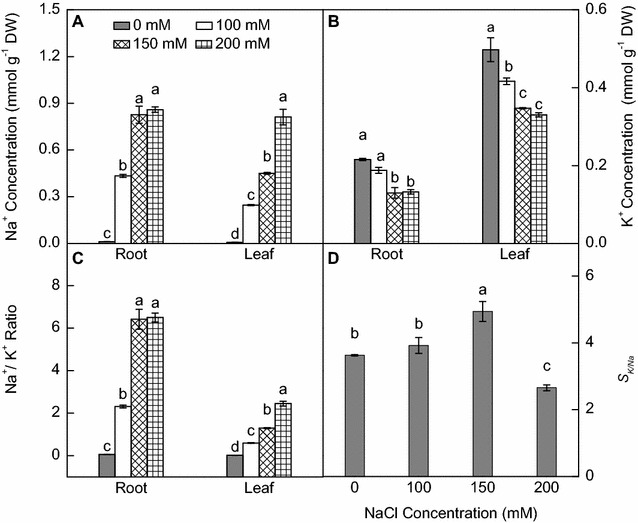
Root and shoot **A** Na^+^ concentration, **B** K^+^ concentration, **C** Na^+^/K^+^ ratio and **D** the ability of ion selective transportation (*S*_*k/Na*_) in wild vetiver seedlings treated with 0, 100, 150, 200 mM NaCl for 18 days. Means (±SE n = 4) followed by *different letters* differ at *p* < 0.05 level

With increasing NaCl levels, wild vetiver plants significantly increased Na^+^ concentration and decreased K^+^ concentration in the roots and leaves (Fig. 5A, B). The Na^+^ concentration was higher in roots than in leaves at 100 and 150 mM NaCl, but both roots and leaves had similar Na^+^ concentrations at 200 mM NaCl (Fig. 5A). Across the NaCl treatments, root K^+^ concentration was significantly lower than leaf K^+^ concentration (Fig. 5B). The Na^+^/K^+^ ratio in the roots and leaves increased with increasing NaCl levels, particularly in roots (Fig. 5C). Compared with the no NaCl treatment, the values of *S*_*k/Na*_ increased at 100 and 150 mM NaCl and reached the highest at 150 mM NaCl, but decreased significantly at 200 mM NaCl (Fig. 5D).

## Discussion

Plants sensitive or tolerant to salinity differ in the rate at which salt reaches toxic levels in leaves, and the time scale may be days or weeks depending on the species and the salinity level (Munns and Tester 2008). Vetiver grass (*Vetiveria zizanioides* L.), a perennial graminaceous plant, is commonly distributed in salt-affected regions, but little is known about the mechanism of its adaptation to salinity. In this study, we examined the salt tolerance of a wild and fertile ecotype of vetiver grass in southern China in terms of plant growth and physiological responses after exposure to a range of NaCl levels for 18 days. We found that this vetiver ecotype was highly tolerant to salinity with little adverse effect on plant growth at 100, 150 mM NaCl, which was probably achieved by a combination of Na exclusion and elevated activities of antioxidant enzymes in leaves.

### Growth response to salt stress

Compared with no NaCl treatment, 200 mM NaCl for 18 days impaired leaf elongation (Fig. 2A) and photosynthesis (Fig. 3A) and reduced relative growth rate by about 44 % (Fig. 2B). In contrast, plant growth was hardly affected by 150 mM NaCl, and the treatments of 100 and 150 mM NaCl even had greater leaf elongation rate than no NaCl (Fig. 2A), suggesting that this wild and fertile ecotype has the ability to maintain plant growth and leaf elongation under highly saline conditions. In a previous study, addition of 100 mM NaCl stimulated both leaf and root growth of a vetiver cultivar (*Vetiveria zizanioides* L. Nash) (Karadge et al. 2011). These findings suggests that moderate Na^+^ can be beneficial to the growth of vetiver grass, a common phenomenon reported in halophytes such as *Atriplex nummularia* (Tester and Davenport 2003) and also in glycophytes such as sugar beet (Wakeel et al. 2010) and barley (Ma et al. 2011), probably for the reason that Na^+^ can substitute for non-specific biophysical functions of K^+^ by maintaining cell turgor especially in stomatal guard cells and ionic balance (Kronzucker et al. 2013; Subbarao et al. 2003).

### Water relation, gas exchange and ion uptake

High external salt concentrations of salts decrease the ability of roots to extract water and high concentrations of salts within the plant itself can be toxic, disturbing many physiological and biochemical processes (Munns and Tester 2008). In this study, leaf *ψ*_*w*_, *WC* (Fig. 1), and *P*_*n*_, *g*_*s*_ and *E* (Fig. 3A, B, D) in vetiver grass was significantly suppressed by 200 mM NaCl, which was consistent with previous reports in millet plants (*Setaria italic L*. and *Panicum miliaceum* L.) (Islam et al. 2011) and bread wheat (*Triticum aestivum* L.) (Kingsbury et al. 1984), but was largely not affected by 150 mM NaCl. The salt-induced osmotic stress would be the primary cause for lowered growth rate at 200 mM NaCl. Under high salinity, once Na^+^ has accumulated to toxic level in plants, plasma membrane depolarization occurs and activation of the outward K^+^ channel can lead to high Na^+^/K^+^ ratio (Cuin and Shabala 2007) and ion-specific toxicity, particularly in leaves (Munns and Tester 2008). Tissue K^+^/Na^+^ ratio is often regarded as a critical trait for salt tolerance in various plant species (Chen et al. 2007; de Souza Miranda et al. 2013; Hauser and Horie 2010; Munns et al. 2012). In this study, increasing NaCl levels increased Na^+^ concentration but decreased K^+^ concentration in the roots and leaves of vetiver grass. Moreover, the roots preferentially accumulated Na^+^ at or below 150 mM NaCl, while leaves preferentially accumulated K^+^ at all NaCl levels (Fig. 5A, B). As a result, the Na^+^/K^+^ ratio in leaves was lowered at or below 150 mM NaCl, but increased at 200 mM NaCl (Fig. 5C). High Na^+^/K^+^ ratio disrupts ion homeostasis and damages plasma membranes (Deinlein et al. 2014). We found that although wild vetiver grass maintained high activity of antioxidant enzymes under saline conditions (Fig. 4), 200 mM NaCl increased MDA production (Fig. 5), an indication of membrane damage. A similar finding was reported in barnyard grass (*Echinochloa crusgalli* L.) (Abogadallah et al. 2010). Low *P*_*n*_ at 200 mM NaCl would be responsible for the increase in MDA through the formation of reactive oxygen species (ROS) which causes membrane lipid peroxidation (Carillo et al. 2011).

Excessive accumulation of Na^+^ in leaves can also cause cation deficiency in K^+^ for example, and inhibits photosynthesis (Gorai et al. 2010). In this study, 200 mM NaCl significantly reduced leaf *P*_*n*_, *g*_*s*_ and *E* in the wild vetiver grass (Fig. 3A, B, D). Leaf *L*_*s*_, a parameter reflecting the degree of stomatal influence on photosynthesis under stress, also decreased with increasing NaCl levels (Fig. 3E), suggesting that the inhibited *P*_*n*_ under salinity was mainly the result of non stomatal limitation.

### Salt tolerance

According to USDA Salinity Laboratory, soils are classified as saline when saturation extract electrical conductivity (ECse) is ≥4 dS m^−1^ (equivalent to 40 mM NaCl), which reduced the yield of most of crops. An ECse of ≥15 dS m^−1^ is considered highly saline (Munns 2005). Growth and physiological parameters can provide reliable criterions for evaluating salt stress or tolerance in plants (Huang et al. 2012; Munns 2005), including changes in length of a growing leaf, plant biomass and leaf stomatal conductance (*g*_*s*_) (Rozema and Flowers 2008). In this study 150 mM NaCl (i.e. 15 dS m^−1^) had no significant effect on leaf *g*_*s*_ (Fig. 3B), *LER* and *RGR* (Fig. 2). In comparison, a previous report (Truong 1994) showed that vetiver grass had a salinity threshold of 8.0 dS m^−1^ and above the threshold yield reduction was 5.26 % per unit dS m^−1^. If the wild ecotype in this study had responded to salinity on a similar scale, its RGR would have reduced by 10, 37 and 63 % at 100, 150 and 200 mM NaCl, respectively. In fact, we measured only 1.3, 9.7 and 44.2 % reductions at the respective NaCl levels (Fig. 2B). The findings may suggest that the wild vetiver grass in southern China has salinity threshold of ~10 dS m^−1^ and is more salt-tolerant than most of common vetiver varieties.

### Possible mechanisms of salt tolerance and prospect for application

Leaves are the main site of Na^+^ toxicity for most plants (Munns and Tester 2008), and therefore maintaining high K^+^/Na^+^ ratio in leaves is essential for plant salt tolerance (Chinnusamy et al. 2005; Munns et al. 2012). Although high K^+^/Na^+^ ratio could be obtained by exclusion of Na^+^ from leaf or acceleration of K^+^ entering into leaf, the ratio was mainly determined by leaf Na^+^ status (Gorham et al. 1987). Increasing evidence indicates that the HKT genes are responsible for retrieval of Na^+^ from the xylem, i.e. the transpiration stream (Munns and Tester 2008). The value of ion selective transportation (*S*_*k/Na*_) is a good measure of retaining Na^+^ in roots, i.e. higher *S*_*k/Na*_ means greater K^+^/Na^+^ discrimination in favor of K^+^ against Na^+^ accumulation in leaves. In this study, the *S*_*k/Na*_ ratio increased at 100, 150 mM NaCl and reached the highest at 150 mM NaCl, indicating that salt tolerance in wild vetiver grass may be largely attributed to Na^+^ exclusion from leaves (NEL) or Na^+^ sequestration in roots. In contrast, previous soil column experiments suggested that high salt tolerance in vetiver was partly due to Na^+^ avoidance by its deep rooting system (NAR) by escaping high salt in the surface soil (Truong 1994) or due to Na^+^ exclusion by root (NER) (Xia et al. 2000).

In the NEL strategy this wild vetiver grass is able to retain a large fraction of Na^+^ in the roots to alleviate the influence of Na^+^ on salt-sensitive leaves, whereas in both NAR and NER strategies plants may exclude or avoid Na^+^ from media around the roots. Undoubtedly, plants in the NEL strategy could absorb more salt from media than plants in the other strategies. Therefore, this wild vetiver grass may have a good prospect in phytoremediation of saline soil and saline water.

In conclusion, our results show that the salinity threshold of this wild vetiver grass is about 10 dS m^−1^, i.e. highly tolerant to salt stress. High ability of K^+^/Na^+^ selective transportation in leaves may be the main strategy for salt tolerance by this wild ecotype of vetiver grass.

## Abbreviations

*LER*: leaf elongation rate
*RGR*: relative growth rate
*ψ*_*w*_: water potential
*WC*: water content
*P*_*n*_: photosynthetic rate
*C*_*i*_: intercellular CO_2_ concentration
*g*_*s*_: stomatal conductance
*E*: transpiration rate
*C*_*a*_: ambient CO_2_ concentration
*L*_*s*_: stomatal limitation value
POD: peroxidase
CAT: catalase
SOD: superoxide dismutase
MDA: malondialdehyde
S_k/Na_: ion selective transportation
